# ‘One Size Does Not Fit All’. The Voices of Professionals Regarding the Delivery of Relationships and Sexuality Education to Children and Young People With Intellectual Disability: Findings From a UK‐Wide Qualitative Study

**DOI:** 10.1111/jar.70223

**Published:** 2026-04-01

**Authors:** Michael Brown, Mark Linden, Lynne Marsh, Maria Truesdale, Fintan Sheerin, Freda McCormick

**Affiliations:** ^1^ School of Nursing and Midwifery Queen's University Belfast UK; ^2^ School of Health and Wellbeing, College of Medical, Veterinary & Life Sciences University of Glasgow UK; ^3^ School of Nursing Maynooth University Kildare Ireland

**Keywords:** children, education, inclusive education, intellectual disability, qualitative research, relationships, sexuality, young people

## Abstract

**Background:**

Relationships and sexuality education (RSE) is critical for children and young people with intellectual disabilities; yet, it is a contentious issue. This study explored the views and experiences of professionals regarding RSE programmes.

**Method:**

Semi‐structured individual interviews were conducted with 16 health, social care and education professionals. Professionals came from across the UK (England *n* = 5, Scotland *n* = 1, Wales *n* = 2, Northern Ireland *n* = 8). Interview data were subjected to thematic analysis.

**Results:**

Three themes emerged: (i) preparation for success; (ii) effective communication and lifelong learning and (iii) individual support and resources. Professionals were motivated and committed to offer the best learning experience possible. There is inconsistency in delivery across programmes, taking time and effort to develop.

**Conclusions:**

The development of structured programmes and tailored resources is necessary to enable practitioners to effectively support and deliver RSE.

## Lay Summary

- This study examined the views and experiences of professionals regarding RSE programme delivery to young people with intellectual disabilities and what helps or hinders the process.
- Semi‐structured interviews were used to collect professionals' experiences of RSE delivery.
- Health, social care and education practitioners were committed to providing children and young people with intellectual disabilities with the best learning experience possible.
- There is a lack of consistency in the content of RSE being delivered by professionals, which often requires considerable preparation time.
- Taking into consideration the vulnerability and safety of children and young people with intellectual disability, RSE should not cease when they leave education at 18/19 years of age.
- There is a need for a structured RSE programme and resources specifically designed for children and young people with intellectual disability.

## 1 Introduction

The delivery of relationships and sexuality education (RSE) to children and young people with intellectual disabilities is critical yet it remains a contentious area within wider society and in special education, health, and social care services (de Wit et al. 2022; Lam et al. 2021). A review by Lam et al. (2021) detailed that the age and gender of members of the public influenced negative attitudes toward issues of sexuality among people with intellectual disability, with males holding more restrictive views. A perception of dependence from some parents and healthcare practitioners, considering people with intellectual disabilities as being ‘eternal children’, were said to result in over protection and anxiety regarding the topic of sexual behaviour (Lam et al. 2021). Despite these views, RSE is increasingly recognised as a fundamental right for all students, including those with intellectual disabilities, given the need to foster self‐awareness, personal safety, and social inclusion (Nelson et al. 2020; UNESCO 2018). Participation in RSE programmes can equip children and young people with the knowledge, positive attitudes, and values necessary to make informed decisions regarding their relationships and sexuality. Lack of RSE places vulnerable children and young people at risk of sexually transmitted infections, unplanned pregnancy, exploitation and abuse (Matin et al. 2021; Stobbe et al. 2021). It is acknowledged by parents, teachers, and young people with intellectual disabilities that relationship and sexuality education is an essential component of comprehensive and high‐quality education. To be a reality, the perspectives of parents, teachers, health professionals, and social care practitioners are essential, as their attitudes, confidence, and strategies shape the quality and inclusiveness of RSE education (Paulauskaite et al. 2022; Brown et al. 2020).

Professionals in these areas of practice often navigate a complex landscape of challenges and opportunities when delivering RSE to students with intellectual disabilities. These include balancing the need for tailored, accessible content with sensitivity to cultural, parental, and institutional expectations (Strnadová et al. 2022; Nelson et al. 2020; Chappell et al. 2018). Moreover, many professionals report limited training in delivering RSE to people with intellectual disabilities, leading to varying degrees of confidence and preparedness (Charitou et al. 2021). Compounding the issues, Canadian researchers identified limited RSE resources suitable for children and young people with intellectual disabilities (Hole et al. 2022). Whilst some professionals strived to find or develop resources, it was recognised that this was highly time‐consuming in an already demanding school environment. This could affect the quality and consistency of RSE delivery among professionals, particularly within the same school (Schmidt et al. 2020). These issues can result in inconsistencies in RSE delivery, with some receiving comprehensive and empowering education while others encounter minimal or inadequate programming. These challenges highlight the need for comprehensive, inclusive, and well‐supported RSE programmes (Pérez‐Curiel et al. 2024).

The current research evidence highlights that the attitudes of teachers, health professionals, and social care practitioners towards RSE for people with intellectual disabilities are influenced by several factors, including personal beliefs, professional training, and perceived support from their respective communities (Deffew et al. 2022). Positive attitudes are often associated with higher‐quality RSE delivery, underscoring the importance of addressing professional concerns and building capacity in this area (Doughty 2021).

Brown et al. (2020) conducted a systematic review of international evidence regarding the design, content, and delivery of RSE programmes for people with intellectual disabilities, offering critical insights into best practices. Their findings highlight the need for RSE programmes that are tailored to the cognitive and social abilities of people with intellectual disabilities, incorporate visual and interactive learning tools, and address key topics such as relationships, consent, and personal safety. The importance of multidisciplinary collaboration among parents, educators, health professionals, and social care practitioners to ensure consistency and inclusivity in RSE delivery is vital for success. Therefore, identifying and addressing the education and development needs of professionals to build confidence and competence in addressing sensitive issues and effectively engaging families and caregivers as active partners in the educational process is required (Strnadová et al. 2022).

There has been a lack of qualitative research on the experiences of professionals involved in the delivery of RSE to children and young people with intellectual disability. Due to their prominent role and wealth of professional experience in working in this field, capturing their lived experiences and professional insights could lead to the development of evidence‐based, tailor‐made resources for these vulnerable children and young people. This study therefore explored the views and experiences of teachers, health professionals, and social care practitioners regarding the delivery of RSE for children and young people with intellectual disabilities, aiming to identify key enablers and barriers in practice.

## 2 Methods

### 2.1 Design

The findings presented here form part of a wider study exploring the views and experiences of children and young people with intellectual disabilities, parents and professionals in the provision of RSE programmes in special schools across the UK (Brown et al. 2023). A qualitative design involving semi‐structured individual interviews was adopted. The interviews collected data on the views and experiences of participants of the delivery approaches regarding RSE for children and young people with intellectual disabilities in special school settings. Due to the scope, depth and extent of the data collected from participants, further detailed analysis from the perspectives of professionals was undertaken (Brown et al. 2023).

Social Learning Theory informed the wider development of the study due to the focus on behaviours and the application of knowledge to real‐world situations (Bandura 1977). To enable the aim of the study to be realised, a pragmatic research paradigm, emphasising methodological flexibility and practical relevance was adopted (Foster 2024). Rather than anchoring the analysis in a predefined epistemological stance, the researchers utilised approaches to effectively address the research questions and supported the generation of actionable insights (Gillespie et al. 2024). This approach reflects a commitment to utility, responsiveness to stakeholder needs, and the integration of different perspectives to inform real‐world application.

### 2.2 Ethics

Ethics approval was granted by the Faculty of Medicine, Health and Life Sciences Research Ethics Committee (ref: MHLS 19_19). All participants were provided with a study information sheet and consent form prior to participating in an interview. All gave written informed consent prior to participation and were informed they could withdraw from the study at any time without giving a reason.

### 2.3 Participants

Participants were recruited from six special schools, two Health and Social Care facilities, and one independent training organisation across England (*n* = 1), Northern Ireland (NI) (*n* = 6), Scotland (*n* = 1), and Wales (*n* = 1). Schools were identified by the research team through information held on the Department for Education website in the four countries of the UK. Schools were randomly contacted until representation was gained from all four countries. Principals were invited to take part in the study or could nominate a colleague. Health and social care professionals were recruited through existing contacts of the research team. The potential participants were then issued letters of invitation and information about the study. All participants had direct experience of RSE delivery.

From February 2022 to February 2023, a total of 16 teachers and health and social care practitioners, collectively referred to as ‘professionals’, took part in the study. A further 11 professionals who were invited to participate or expressed interest were either unable to attend an interview, *n* = 4, or, following contact on several occasions, did not respond, *n* = 7. Table 1 details the role and geographical locations across the United Kingdom.

**TABLE 1:** Role and geographical location of professionals.

Professional identifier	Role	Region
1	Teacher	England
2	Teacher	Wales
3	Teacher	Wales
4	Teacher	Northern Ireland
5	Teacher	Northern Ireland
6	Teacher	Northern Ireland
7	Teacher	England
8	Teacher	Northern Ireland
9	Teacher	Northern Ireland
10	Teaching assistant	England
11	Registered nurse	England
12	Registered nurse	Northern Ireland
13	Manager	England
14	Manager	Northern Ireland
15	Manager	Northern Ireland
16	Manager	Scotland

### 2.4 Data Collection

Individual semi‐structured interviews took place with 16 health, social care and education practitioners, with each lasting between 26 and 66 min. Interviews took place via Microsoft Teams, telephone, or in person in the professional's school. Each interview was conducted by the same author for consistency (F.M.).

Interview questions centred on the participants' views on and experiences of RSE programmes: how is RSE currently delivered in your schools? What are the barriers or facilitators to bringing RSE programmes into schools? What has worked well when teaching about RSE in school? What topics would you deem necessary to be included in RSE programmes? What do you think are the best methods to teach young people with intellectual disability about RSE? and how important is it to learn about relationships and sexuality in school?

All interviews were recorded and transcribed *verbatim* and anonymised with the removal of all identifiable information. Each participant was assigned a numerical identifier.

### 2.5 Data Analysis

Interview transcripts were read, re‐read and analysed by two members of the research team (M.B. and F.M.) in accordance with the six‐phase approach by Braun and Clarke (2006) to gain an understanding of the participants' views and experiences. These procedures included familiarisation with the data, generation of initial codes, searching, reviewing and defining themes and creating the report. All data were coded into categories with the analysis facilitated by the data management programme NVivo 12 (Lumivero 2025). Themes were systematically identified and thoroughly discussed by M.B. and F.M. Any disagreements were resolved through reflection on individual positionalities and open dialogue. If disagreements were to occur, a third member of the team (M.L.) offered an independent perspective. Disagreements were resolved between M.B. and F.M. Themes were shared with the research team who provided final oversight and approval. Throughout the data analysis and synthesis process the approaches used were rigorously followed to ensure trustworthiness, transferability, dependability, confirmability and credibility (Lincoln and Guba 1985). M.B. and F.M. re‐read the transcripts on multiple occasions and brought their themes to the wider team for checking. Descriptions of participants and their contexts are provided above to enable readers to judge how transferable findings are to their own situations. Using an established procedure for data analysis, documented via NVivo 12 ensured dependability of the process. Lastly, inclusion of multiple participant quotations was employed to ground analysis in their lived experience and confirm findings.

The research team comprised registered nurses, psychologists, and researchers working in Northern Ireland, Scotland, and the Republic of Ireland. We were aware that our combined interest in the topic under investigation, our professional backgrounds, and individual contexts may have influenced our approach to the research and analysis. To mitigate these factors the team met on a regular basis to discuss ethical procedures, interview questions, recruitment and analysis with the purpose of challenging any in‐grained assumptions we might hold. The researcher (F.M.) conducting the interviews sought to establish rapport and was aware of the inherent power dynamic when conducting an interview. The researcher provided space for participants to guide the conversation within the confines of a semi‐structured interview; participants were given control as to whether their cameras were on or off; were reminded that the conversation would be held in confidence; and acknowledged some of the inherent limitations of online interviews, that is, reduced recognition of nonverbal cues and emotional connection, connectivity issues.

## 3 Results

Most professionals were teachers (*n* = 10) with four managers and two registered nurses. Eight professionals were based in Northern Ireland, with five from England, two from Wales, and one from Scotland. The analysis of the views and experiences of professionals regarding the development and delivery of RSE programmes is presented under three themes: (i) preparation for success; (ii) effective communication and lifelong learning and (iii) individual support and resources.

### 3.1 Theme 1: Preparation for Success

Theme 1 identified how professionals established rapport and built confidence in children and young people, viewed as essential for success. The professionals detailed the satisfaction gained through preparation and the provision of support that resulted in positive engagement in RSE programme delivery. The professionals recognised their privileged role and the significance of RSE for the development and futures of the children and young people with intellectual disabilities. They appreciated that the effective delivery of the RSE programme had the potential to positively impact on and influence the lives of the children and young people:

We've got such a privileged position that we're actually going to be enabling them to keep safe. Know when something's right, when something's wrong and then, how to then tell somebody if they don't feel safe, or to experience love. All of our children deserve to feel loved in whichever capacity, whether it's a friendship, it's a relationship. (Professional 11)

Staying up to date with current RSE issues, particularly those highlighted in the media, was seen as important. It was also viewed as an opportunity to introduce and discuss potentially controversial issues and concerns with the children and young people:

Online relationships and things like that are becoming more prevalent and it's important that we adapt to that and include that in. (Professional 5)

We do look at the online world and what that means. We've shattered many illusions in reality and TV and stuff. (Professional 14)

When preparing RSE lessons, the professionals aimed to future‐proof the content, ensuring the information was relevant not only to the young person's current stage of life and needs while supporting the transition into the responsibilities of adulthood:

We'll give them the tools that they need and give them the awareness and understanding of what is right and wrong and what is appropriate for them to do … Because obviously it can be done to them in the future … I said it to pupils from my class before, things like that could be done to them and is this acceptable, that kind of thing. (Professional 7)

It's having the courage to actually stand up and say no, I don't want this. Because sometimes our children can come across as a lot more understanding and capable than they are, and then they could be in a situation that they don't know how to get out of. (Professional 10)

In the early stages of RSE programme delivery, it was viewed as crucial to take the time to build rapport and confidence with the children and young people. This was seen as essential in fostering openness and reducing embarrassment for both the young people and professionals. The use of real‐life examples and tangible objects was also considered important, as it helped the students to more fully engage with and better understand the lesson content and the learning materials:

Generally, those first couple of sessions are like ease in sessions. Like something that's not too much for them to handle initially. So, they get comfortable and then you can start to explore those kinds of deeper things a little bit … Be very open and honest about how I'm feeling too. And I think that helps because that allows some of the pupils to relate to me delivering it. But it can be a bit embarrassing for me too, but it's important that we talk about things, and we keep things very open in that regard. (Professional 4)

What worked well, was always having as realistic things as possible for them to look at. They're not very, some of them aren't very great at using their imagination, or you want a life‐like vagina and penis and everything. (Professional 1)

The professionals emphasised the importance of dedicating time to prepare for each session and the need to source and develop appropriate learning resources. However, this required significant time, and suitable materials were not always readily available.

### 3.2 Theme 2: Effective Communication and Lifelong Learning

Theme 2 highlighted the need for inclusive, paced, and tailored delivery of RSE, recognising the importance of effective communication to build trust, thereby seeking to address diverse needs. This was viewed as necessary to enable and support confident, respectful engagement and reduce vulnerability. Professionals were aware of the importance of delivering RSE information and the range of approaches and strategies needed to clearly and effectively communicate with the young people, given their individual and distinct needs:

Back in the day, you got the talk about the birds and the bees, but now it seems to be much more sensible, would you say. I mean there's no point talking to our kids about birds and the bees and then they go and they're going, right I've no clue what that was about, you know, but if you just say openly what it's about and stuff. (Professional 9)

I think everything's down to being able to communicate effectively with that individual and learning what their communication styles are and what works best for them. I think you're going to be able to best deliver that education to them because if you're just doing kind of one size fits all and delivering it in one way, I don't think that's going to work. (Professional 12)

Participants were mindful of the range of relationship and sexuality issues to be covered in the curriculum and of the importance of delivering at an appropriate pace and depth of content. It was viewed as crucial to effective learning to ensure that the essential principles were grasped and understood before moving on to focus on more detailed information:

It's pitching it at that right level, isn't it, and understanding first the basics of, you know, friendships and relationships and those barriers around that before you can even think about that sort of sex education. (Professional 13)

It was acknowledged that young people with intellectual disability were often left out of discussions about relationships and sexuality, and may not have the chance to continue conversations at home after taking place in school:

Young people without a learning disability, parents are a little bit more flexible in having those conversations. I think people with an ID [intellectual disability] would quite often find parents and carers resistant to having those conversations. So, if they want to go home to talk about it further, they can't. They have barriers. (Professional 16)

Moreover, this could place them in potentially vulnerable situations, as they may not appreciate that some of their comments could constitute hate crimes or homophobia:

They've never had those discussions about what is different … there'll be lesbian and gay men, there'll be trans people, and I will invite them in, and they will meet them. And they can ask all those questions. And I think that will help with the hate crime stuff and things as well … knowledge is power and we need to introduce folk to people so it's a real, tangible person that they can now think about … or if they've got their own questions about their own gender, then they can have that conversation. (Professional 16)

The professionals experienced a sense of satisfaction and achievement as the young people gained confidence and deepened their knowledge and understanding of relationships and sexuality issues. The trust and rapport built over time fostered engagement, encouraging the young people to participate in discussions and ask questions:

One of the positives is, seeing as each lesson goes on, the pupils becoming more open and comfortable with having the lessons and the conversation starts to go on … it's important that it comes from us, those that they can trust. (Professional 5)

However, despite the substantial effort professionals invested in teaching and communicating with the young people in their care, there were concerns that RSE ended upon leaving school, with on‐going communication and information sharing channels no longer existing:

They leave us and they are quite cloistered here. It's like a family atmosphere and everybody knows them and they know who to talk to and they feel really open and comfortable to talk about anything … And then, they leave us. And there is very little post‐19 provision. The provisions are very scarce on the ground. (Professional 6)

### 3.3 Theme 3: Individual Support and Resources

Theme 3 highlighted that RSE delivery needs to be individualised and continuous. Effective delivery involved adaptive approaches that support reinforcement, individualised delivery, and appropriate resources, yet some professionals experienced significant challenges due to limited evidence‐informed guidance and resources. Relationship and sexuality education was viewed not as a one‐time series of lessons, rather a continuous on‐going process throughout life. There was a need for reinforcement and repetition with the clear understanding that a one‐size‐fits‐all approach will not meet the needs of these children and young people. Repeating RSE content from previous lessons was seen as essential to reinforce understanding and ensure the young people grasped the material and the opportunity to build on and develop learning:

And again, just repetition, repetition. (Professional 8)

Just repeat, repeat, repeat … Over and over and over again. And trying every which way you can until it finally works. (Professional 6)

What works well as well is that reinforcement … it's just reinforcing the messages and the information again. (Professional 15)

The success of continuous reinforcement over the years was evident while also leading to additional challenges for the professionals, who felt responsible for supporting the young people with any information they were willing to share or seek:

I just think they're unbelievable and all these years later they still just blow my mind. The discussion that they generate and the questions that they ask me. They're just so aware and so up to date and you just can never under‐estimate even their cognitive ability that you think you know what they actually do know about sexuality … And it's terrifying, don't get me wrong, I'm absolutely terrified all these years later still teaching the subject. (Professional 6)

Whilst the professionals adhered to curriculum requirements and guidelines when preparing RSE lesson content, some experienced challenges in tailoring it to the needs of individual students and those of the wider class:

We follow the kind of guidelines and what you should cover but it's very hard making that in a way that is valuable and interesting to our young people. It's hard to know…because usually curriculums follow age, don't they. So, when we're looking at pupils' ability, and then to put it into the mix, the kids aren't necessarily in year groups in our school. So, you might be teaching a topic, um, sex, and you've got some year sevens and some kind of 15‐year‐olds at the end of key stage four. So, it's generally difficult, our PSHE curriculum. (Professional 1)

A primary concern shared across the professionals was the inadequate availability of resources tailored to meet the needs of children and young people with intellectual disability:

We are limited in what resources are available, especially for more of those with sort of sensory needs … it's definitely a challenge and we're sort of finding our way through it to get appropriate resources that are going to cater for everybody really … I think it's the resources side of things and the limitations there of resources to meet every individual's needs and it's trying to get together and get ideas for that as well. (Professional 5)

The materials sometimes maybe aren't as accessible to our young learners. (Professional 8)

Furthermore, there was no evidence‐informed, structured programme or lesson plans and evaluations, leaving professionals to determine what to teach and when and how to determine the effectiveness of delivery and implementation:

It's where do you start, and should religion or ethnicities come into when you're delivering. As professionals we're delivering what we consider is safe lessons and safety for your children … we're acting on the children's welfare … we should do what we feel is best for the children. (Professional 2)

At the minute things feel like, it's everywhere, there's no structure. So, ideally what I would like is a programme where it has term one, you teach this, term two, it is this, term three, it's this, that's year one. Then what do we do in year two. So, it's very structured. Us teachers like structure. (Professional 8)

For all the professionals, it was essential to equip and prepare the young people with the necessary skills, knowledge and resources to enable effective learning, thereby ensuring they understood the concepts by the time they left school, with the potential to apply them in their adult lives. One professional likened their experience of teaching RSE to preparing troops for war.

I think it's a bit like going to war. You learn how to shoot a rifle and you learn how to run about the place, but you don't actually use that until you go to war. It's a bit like that…we are training our pupils up to have the resources and the skills that they need, and they probably won't use those skills to a large extent in school and will then start using them the moment they leave school. (Professional 9)

It was emphasised that RSE should continue for young people with intellectual disability after they leave special education and should be offered throughout adult and older people services. This would reinforce and help retain the knowledge and skills they gained in school, making them more relevant as they navigate life and relationships beyond school, on into adulthood:

It can't stop, because whilst people are having relationships, it can't stop. We've got to keep it open and a rolling programme. (Professional 16)

## 4 Discussion

RSE is a policy requirement within the school curriculum providing general guidance for classroom delivery across the United Kingdom (Department for Education (DfE) 2025). An Australian systematic review identified the primary aim of RSE programmes is to provide children and young people with core knowledge and understanding to support their navigation through the complexities of friendships, relationships and the expression of sexuality, thereby shaping their attitudes, values and beliefs (van Leent et al. 2023). Ensuring that children and young people with intellectual disabilities have access to evidence‐informed RSE programmes, specific to their learning needs is necessary (Carter et al. 2021). Failure to recognise and address their needs place them at the potential risk of harm, exploitation, abuse, unintended pregnancy and sexually transmitted infections (Matin et al. 2021; Stobbe et al. 2021). To address these needs, the professionals in the current study were motivated and committed to providing the children and young people with a positive learning experience relevant to their individual needs. Their commitment included seeking out focused lesson content and resources tailored to needs and level of understanding and comprehension of individual and groups of students (Crehan et al. 2023). Yet, professionals highlighted the need for evidence‐informed guidance and RSE programmes, with appropriate learning resources, a position evidenced in the systematic review conducted by Paulauskaite et al. (2022). Without evidence‐informed programmes, the ability to evaluate and identify the benefits of RSE, a finding reiterated across systematic reviews (Brown et al. 2020; Paulauskaite et al. 2022).

It was evident some professionals were more confident in their approach to lesson planning and delivery than others, findings aligning with those identified in a Swedish study (Nelson et al. 2020). Integrating topics relevant to current media stories and social events into some programmes was recognised as relevant and significant to ensure content was contemporary and current. This was achieved by embedding real‐life examples as scenarios and discussion points, thereby helping to ensure that learning became more meaningful and relevant (Roden et al. 2020). This allowed for greater awareness of consequences and the development of practical strategies that had the potential to be generalised to similar situations. Many children and young people with intellectual disability are vulnerable to exploitation, harm and abuse, with the professionals recognising their responsibility and the lasting impact on the lives of their students (Svae et al. 2023; Idle et al. 2025). It was acknowledged that this influence extends beyond the classroom, fostering openness for future conversations and confiding. Australian researchers concluded that having a ‘trusted adult’ in their lives potentially avoids seeking information from external sources which could be inaccurate and misleading (Devkota et al. 2025).

Arising from the findings from the current study are implications for policy, practice, education and future research. The past decades have seen major policy shifts regarding the support of people with intellectual disabilities, with moves away from institutional models of care to community‐based, aiming to promote their social inclusion (Louw et al. 2020). Consequently, people with intellectual disabilities are visible within communities, seeking the same rights and opportunities as all citizens (Buchner et al. 2021). From this is the need to ensure that their distinct needs, including those related to relationships and sexuality, are recognised within policies, resourced, planned for and responded to. A policy failure to do so will increase and not reduce the inequalities gap, with significant implications for people with intellectual disabilities and their families (Nair et al. 2022). Guidance from the UK Department for Education for typically developing pupils suggests several key areas be addressed including Health and Wellbeing, LGBT+, Law, Equality and Religion. The guidance on pupils with special educational needs (SEND) spans two paragraphs and suggests subjects should be made accessible and that teachers should be made aware of the increased vulnerabilities of SEND pupils. The Sex Information and Education Council of Canada based their guidance on sexual health education around nine core principles. The principles emphasise the importance of evidence‐informed, developmentally appropriate instruction that is inclusive of diverse identities and experiences, including those of individuals with disabilities. Central to the framework is the promotion of healthy relationships, critical thinking, and personal agency, ensuring that learners are equipped to make informed decisions about their sexual health. The guidelines advocate for culturally responsive pedagogy delivered by competent educators, underscoring the need for sustainable integration of sexual health education within broader educational systems.

Implications for education include the requirement that teachers, nursing students, and other professionals undertaking professional preparation are provided with foundational RSE education and training as part of their programmes to act as the building blocks to their future practice (Chrastina and Večeřová 2020). There is further opportunity at post‐graduate level to develop inter‐professional RSE to enable growth and depth of understanding regarding the development, implementation and evaluation of programme delivery and the outcomes and benefits achieved (Paulauskaite et al. 2022). To promote the on‐going development of practice, the establishment of local, national and international networks, and the provision of opportunities to shadow other professionals could enable the sharing of best practice and future interprofessional collaborations (Schot et al. 2020). This has the potential to increase confidence, knowledge and skills of practitioners across organisations. Developments in practice are necessary to support the on‐going development and delivery of RSE programmes to children and young people with intellectual disability, notably those with more complex needs, to equip them with knowledge, support and skills and the attitudes and values necessary to address the opportunities and challenges of life (Schalock et al. 2021).

While the research evidence‐base in RSE is evolving and growing, there is scope for further enquiry. Despite the apparent need, concerns were identified in the current study that RSE programmes typically end when young people leave school, with limited continuity or opportunity for reinforcement in adult services or further education and development. This is a gap that needs to be researched and addressed for the future as the population of adults and older adults with intellectual disabilities with complex needs age. There is an opportunity to conduct multi‐centre and longitudinal studies following up groups of young people with intellectual disabilities as they transition into adulthood to identify the retention and application of their RSE learning of the realities of friendships and relationships. Studies are also required that identify the impact of participating in RSE and outcomes achieved in areas such as a reduction in safeguarding referrals, a decrease in unintended pregnancy and sexually transmitted infections. There is also scope to research focusing on the impact and outcomes of interprofessional education that develops knowledge and skills in RSE programme development and delivery.

### 4.1 Strengths and Limitations

A strength of the current study is the inclusion of participants drawn from a range of settings including special schools, healthcare facilities and private training providers, all with expertise in the delivery of RSE programmes specific to the needs of children and young adults with intellectual disabilities. The participants were from different professions, adding a wider dimension and understanding of the contrasting perspectives regarding their experiences of RSE delivery and the contributions made. While rigorous attempts were made to recruit participants from across services and professional backgrounds, it is acknowledged that not all groups involved in RSE programme delivery are represented. Therefore, the views and experiences of the study participants may not be reflective of those of professionals delivering RSE programmes in other contexts and settings. Many of the final study participants were from Northern Ireland, with fewer recruited from Wales and Scotland. The researchers acknowledge this as a potential limitation that may affect the transferability of the findings. Several potential participants (*n* = 11) initially expressed interest in taking part in the study, subsequently opting not to take part or were uncontactable. It was not possible to identify their reasons for non‐participation, which may indicate a degree of non‐response bias.

## 5 Conclusions

This study presents the findings of the in‐depth views and experiences of professionals involved in the delivery of RSE to children and young people with intellectual disability. The professionals who participated detailed the need for dedication and on‐going commitment in the preparation and delivery of RSE programmes. Professionals require dedicated time to prepare RSE programme delivery and the identification of resources specific to the needs of individual students. This is necessary to ensure students are equipped with the necessary knowledge and skills and attitudes and values, necessary to navigate life safely in society as they transition into adulthood. To enable delivery, communication between the professionals involved in RSE programme delivery is needed to ensure information is shared. For RSE delivery to be effective, it is necessary for programmes to be tailored to the distinct needs of children and young people with intellectual disabilities, supported by tailored resources. This could potentially ease the burden for professionals in the delivery and support of RSE. Whilst there would be associated economic costs in the creation of learning resources, an evidence‐based and rigorously tested programme that could be delivered at scale would reduce costs and improve RSE outcomes specific to the distinct needs for these children and young people. Future research by way of multi‐centre and longitudinal studies could examine how children and young people with intellectual disability retain knowledge and skills obtained through RSE participation and apply their learning in real‐world situations as they transition into adulthood.

## Author Contributions

M.B. and M.L. designed the study. F.M. collected the data. M.B., M.L., L.M., M.T., F.S. and F.M. analysed the data. M.B., M.L., L.M., M.T., F.S. and F.M. wrote the manuscript. All authors read and approved the final manuscript.

## Funding

This study was funded by The Burdett Trust for Nursing. For the purpose of open access, the authors have applied a Creative Commons Attribution (CC BY) licence to any author‐accepted manuscript version arising from this submission.

## Conflicts of Interest

The authors declare no conflicts of interest.

## Data Availability

The data that support the findings of this study are available from the corresponding author upon reasonable request.
